# White matter alterations and their associations with biomarkers and behavior in subjective cognitive decline individuals: a fixel-based analysis

**DOI:** 10.1186/s12993-024-00238-x

**Published:** 2024-05-22

**Authors:** Yi-Chia Wei, Yi-Chia Kung, Ching-Po Lin, Chih-Ken Chen, Chemin Lin, Rung-Yu Tseng, Yao-Liang Chen, Wen-Yi Huang, Pin-Yuan Chen, Shin-Tai Chong, Yu-Chiau Shyu, Wei-Chou Chang, Chun-Hung Yeh

**Affiliations:** 1 Department of Neurology, Chang Gung Memorial Hospital, Keelung, 204 Taiwan; 2 Community Medicine Research Center, Chang Gung Memorial Hospital, Keelung, 204 Taiwan; 3 College of Medicine, Chang Gung University, Taoyuan, 333 Taiwan; 4 Institute of Neuroscience, National Yang Ming Chiao Tung University, Taipei, 112 Taiwan; 5 Department of Radiology, Tri-Service General Hospital, National Defense Medical Center, Taipei, 114 Taiwan; 6 Department of Education and Research, Taipei City Hospital, Taipei, Taiwan; 7 Department of Psychiatry, Chang Gung Memorial Hospital, Keelung, 204 Taiwan; 8 Department of Medical Imaging and Radiological Sciences, Chang Gung University, Taoyuan, 333 Taiwan; 9 Department of Radiology, Chang Gung Memorial Hospital, Keelung, 204 Taiwan; 10 Department of Neurosurgery, Chang Gung Memorial Hospital, Keelung, 204 Taiwan; 11 Department of Nursing, Chang Gung University of Science and Technology, Taoyuan, 333 Taiwan; 12 Department of Psychiatry, Chang Gung Memorial Hospital at Linkou, Taoyuan, 333 Taiwan

**Keywords:** Subjective cognitive decline, Fixel-based analysis, Diffusion MRI, Blood biomarker, Axonopathy, Amyloid, Superior longitudinal fasciculus

## Abstract

**Background:**

Subjective cognitive decline (SCD) is an early stage of dementia linked to Alzheimer's disease pathology. White matter changes were found in SCD using diffusion tensor imaging, but there are known limitations in voxel-wise tensor-based methods. Fixel-based analysis (FBA) can help understand changes in white matter fibers and how they relate to neurodegenerative proteins and multidomain behavior data in individuals with SCD.

**Methods:**

Healthy adults with normal cognition were recruited in the Northeastern Taiwan Community Medicine Research Cohort in 2018–2022 and divided into SCD and normal control (NC). Participants underwent evaluations to assess cognitive abilities, mental states, physical activity levels, and susceptibility to fatigue. Neurodegenerative proteins were measured using an immunomagnetic reduction technique. Multi-shell diffusion MRI data were collected and analyzed using whole-brain FBA, comparing results between groups and correlating them with multidomain assessments.

**Results:**

The final enrollment included 33 SCD and 46 NC participants, with no significant differences in age, sex, or education between the groups. SCD had a greater fiber-bundle cross-section than NC (*pFWE* < 0.05) at bilateral frontal superior longitudinal fasciculus II (SLFII). These white matter changes correlate negatively with plasma Aβ42 level (*r* = −0.38, *p* = 0.01) and positively with the AD8 score for subjective cognitive complaints (*r* = 0.42, *p* = 0.004) and the Hamilton Anxiety Rating Scale score for the degree of anxiety (Ham-A, *r* = 0.35, *p* = 0.019).

The dimensional analysis of FBA metrics and blood biomarkers found positive correlations of plasma neurofilament light chain with fiber density at the splenium of corpus callosum (*pFWE* < 0.05) and with fiber-bundle cross-section at the right thalamus (*pFWE* < 0.05). Further examination of how SCD grouping interacts between the correlations of FBA metrics and multidomain assessments showed interactions between the fiber density at the corpus callosum with letter-number sequencing cognitive score (*pFWE* < 0.01) and with fatigue to leisure activities (*pFWE* < 0.05).

**Conclusion:**

Based on FBA, our investigation suggests white matter structural alterations in SCD. The enlargement of SLFII's fiber cross-section is linked to plasma Aβ42 and neuropsychiatric symptoms, which suggests potential early axonal dystrophy associated with Alzheimer's pathology in SCD. The splenium of the corpus callosum is also a critical region of axonal degeneration and cognitive alteration for SCD.

**Supplementary Information:**

The online version contains supplementary material available at 10.1186/s12993-024-00238-x.

## Introduction

### Subjective cognitive decline (SCD)

Subjective cognitive decline (SCD) is the early stage of cognitive impairment and is defined as self-awareness of declining memory or other cognitive abilities relative to their previous level of performance in the absence of objective neuropsychological deficits [1]. Multiple aspects are involved in SCD when comparing to normal aging populations, like increased anxiety and depression tendency, impaired sleep quality, physical inactivity, and reduced quality of life, along with the increase in subjective cognitive complaints [2]. Researchers have recently turned to SCD as an effective tool for detecting the early stages of Alzheimer's disease (AD). SCD is believed to mirror the initial cognitive decline associated with AD, and studies have proven that individuals who report SCD are at a higher risk of developing AD compared to those who do not report any cognitive decline [3].

Research has shown that people with SCD display comparable pathological patterns to individuals with early AD, including the accumulation of amyloid beta and tau protein in the brain [4–6]. People with SCD have shown genomic modifications involving the pathways of beta-amyloid (Aβ) metabolism [7]. Additionally, studies on neuroimaging have shown that SCD is linked to both functional and structural changes that occur early in the AD continuum [8, 9]. Therefore, SCD is considered a transitional phase between normal status to early AD [1, 10]. The growing body of research on SCD is helping to shed light on early AD and may ultimately lead to improved early detection and treatment [11, 12].

Diffusion MRI (dMRI) has been applied to investigate changes in white matter pathways that are associated with SCD [8, 9]. Diffusion tensor imaging (DTI) revealed that individuals with SCD recruited from memory clinics and communities showed white matter alterations, particularly in the corpus callosum [13–18], superior longitudinal fasciculus (SLF) [15, 17–19], corticospinal tract [15–17, 20], thalamic radiation [15–17], cingulum [13, 16, 18, 20], and hippocampus [13, 17, 21]. While in these studies, DTI metrics often showed lower fractional anisotropy (FA) and higher mean diffusivity (MD) than normal controls; additionally, radial diffusivity [22] and axial diffusivity [15] could also increase. Despite some studies showing nonsignificant or borderline results in group comparisons between SCD and NC [23], it is typically observed that the values of the DTI metrics of SCD fall between those of NC and mild cognitive impairment (MCI)/AD [23–25].

### Fixel-based analysis (FBA)

Since white matter variations in SCD likely occur in the early neurodegenerative process, the subtle changes might reveal the initiation mechanism of cognitive decline in the dementia continuum. However, the conventional technique applied to analyze dMRI data of SCD, mainly tract-based spatial statistics (TBSS) using DTI [26], is known to have limitations in providing accurate white matter measures in voxels with complex fiber arrangements; this induces uncertainties and poses challenges interpreting the results [27–29]. Therefore, there is an urgent need for more sophisticated and sensitive techniques to identify the earliest white matter alterations in the dementia continuum.

FBA is an advanced technique that can estimate white matter fiber-specific measures from dMRI data for group analysis [30]. In FBA, fixels can be derived from fiber orientation distributions (FODs) of each voxel as typically computed from constrained spherical deconvolution [31, 32]. Each fixel represents an element of the fixel grid that contains information on specific fiber orientation within an individual voxel [30]. The fixel-wise parameters include microstructural fiber density and macrostructural fiber-bundle morphology; they can reflect direct features of white matter fiber alterations such as axonal loss or atrophy, respectively [33]. In addition, FBA utilized a population template generated based on the study cohort, rather than transforming imaging data to an MNI template in common voxel-based approaches; such a study-specific template can be more representative of the study population [26].

Because of the advantages in detecting axonal changes, FBA has been used in studying the AD continuum in previous studies [34, 35]. Dewenter et al. enrolled the full spectrum of biomarker-confirmed AD and amyloid- and tau-PET negative controls. The results showed weak associations of fixel metrics with amyloid and tau depositions in the brain. Both the density and cross-section of fiber bundles decreased as amyloid deposition increased, but there was no additional decline due to abnormal tau deposition [34]. Mito et al. used FBA to compare the white matter loss in AD, MCI, and healthy controls. Both microstructural and macrostructural white matter loss are noticed in AD patients associated with default mode network nodes, but the reduction of density and cross-section of fiber bundles are limited in the posterior cingulum in MCI when compared with healthy controls [35]. These studies highlight the potential of using FBA in revealing early axonal morphology differences in the dementia continuum. One of the earliest morphological changes associated with AD is known to be axonal swelling or dystrophy in the brain [36, 37], which was however not observed in the previous work. In this study, FBA is used to study SCD, which is the earliest stage of the AD continuum before the MCI stage. Leveraging the advantages of FBA, the current study aimed to evaluate our hypothesis that axonal dystrophy may occur in the stage of SCD and may correlate with blood biomarkers and clinical assessments.

### Blood biomarkers of SCD

Identifying effective biomarkers is the current trend in studying AD, and the concepts evolved from syndromal diagnosis to a biological definition of AD [38, 39]. In addition to molecular imaging and cerebrospinal fluid detection [40], novel developments have been expanded to multiple high-sensitivity blood biomarkers for early AD detection. Plasma beta-amyloid 42 (Aβ42), beta-amyloid 40 (Aβ40), total tau protein (Tau), phosphorylated tau 181 (p-Tau181), neurofilament light chain (NfL), and glial fibrillary acidic protein (GFAP) are considered alternative approaches to confirm the biological pathogenesis of AD [41, 42]. Plasma biomarkers have the ability to detect early and preclinical pathological changes in AD. A study at a memory clinic discovered notable differences in plasma p-Tau181 and NfL levels between patients with AD, MCI, and SCD. The ratios of Aβ42/Aβ40 and p-Tau181/Aβ42 also varied significantly among the different patient groups [43]. Furthermore, the level of plasma amyloid can indicate early-stage amyloid pathology in the brain. Plasma Aβ42 level and Aβ42/Aβ40 ratio showed a good correlation with cerebrospinal fluid Aβ42 in SCD [44].

Additionally, individuals with SCD have an increased presence of the cytotoxic form of Aβ in their plasma compared to normal controls. That is, Aβ in AD pathogenesis exhibits polymorphic patterns, and amyloid-beta oligomer (AβO) is the most cytotoxic soluble form of Aβ that causes neuronal injuries since the beginning of AD pathogenesis [45]. Kim et al. conducted a study to examine the relationship between plasma AβO and subjective cognitive complaints in individuals with normal objective cognition. They found correlations between the plasma concentration and the ratio of AβO to the degree of subjective cognitive complaints. Besides, a high level of AβO was found to associate with brain amyloid deposition in a study based on [^18^F] flutemetamol positron emission tomography (PET) [46].

Plasma Aβ is also linked to the change of brain white matter in preclinical dementia. According to the TBSS analysis of dMRI data in the Sino Longitudinal Study on Cognitive Decline (SILCODE) , lower FA and higher MD values appeared in the widespread white matter of both hemispheres in SCD individuals with a higher plasma Aβ40 [9]; it was the first time plasma biomarkers and dMRI were combined in studying SCD. However, the amyloid-associated brain regions did not showregional specificity or provide further implications of neurodegenerative mechanisms. The brain-behavior/cognition and biomarker-behavior/cognition associations were also lacking.

### Study aims

This study leverages the capability of FBA to investigate white matter fiber changes in preclinical dementia, by comparing adults with SCD to healthy controls. In addition, the FBA metrics are further analyzed dimensionally alongside five other domains of assessments; they are blood-based biomarkers of neurodegenerative proteins and multidomain behavior assessments, including cognitive, mental, physical activity, and fatigability examinations.

## Methods

### Participant enrollment and grouping

The Community Medicine Research Center of the Chang Gung Memorial Hospital in Keelung launched the Northeastern Taiwan Community Medicine Research Cohort in 2012 (NTCMRC, NCT04839796 on ClinicalTrials.gov). Our participant enrollment was joined with the community cohort study during 2018–2022.

All the participants had neuropsychiatric assessments, cognitive tests, blood tests, and brain MRI scans. The participants were excluded from the present study when they had: (1) a history or active state of psychiatric diseases found in the Mini-International Neuropsychiatric Interview (MINI) (see next section), (2) impaired cognitive performance when their Montreal Cognitive Assessment (MoCA) score (see next section) was lower than one standard deviation below the mean of age- and education-stratified norms [47], (3) a history of brain disorders like traumatic brain injury, stroke, brain tumor, and cranial surgery, (4) major organ failure, (5) lesions on structural brain MRI images or (6) inadequate image quality found in brain MRI or during image preprocessing.

The cognitively and psychiatrically normal participants were queried for self-reported subjective cognitive complaints (SCCs) by the Eight-item Informant Interview to Differentiate Aging and Dementia (AD8) questionnaire [2, 48–50]. A score of AD8 ≥ 2 points (AD8 total score ranged from 0 to 8) was considered a confirmation of having SCCs [48, 50]. After completing the AD8 report, those with SCCs were grouped into the SCD group. The participants with no SCCs were in the normal control (NC) group.

**Fig. 1:**
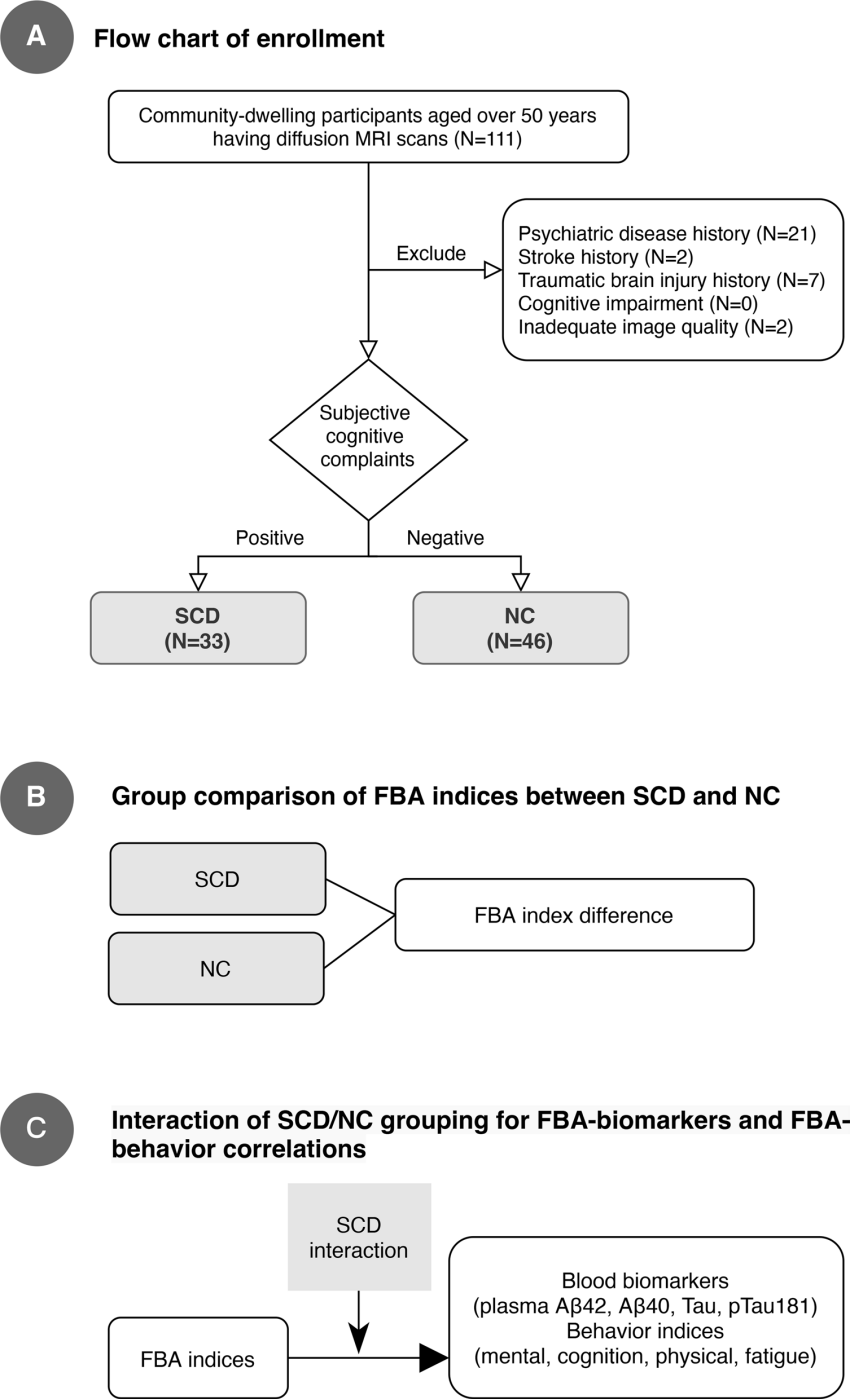
Overview of the study design. **A:** A total of 111 middle-aged and older adults from the communities were screened for eligibility, and 32 were excluded. The final enrollment was divided into subjective cognitive decline (SCD, N = 33) and normal control (NC, N = 46) by having or not having subjective cognitive complaints defined by an AD8 score of 2–8 or 0–1 points, respectively. **B:** We first compared the FBA metrics between SCD and NC groups. **C:** We tested the interaction of SCD grouping between FBA-biomarkers and FBA-behavior correlations

Figure 1 provides the general workflow of the current study. Of the 111 participants undergoing initial screening, there were 21 with psychiatric disease history, seven with traumatic brain injury history, and two with stroke history (Fig. 1A). The participants were grouped into SCD and NC groups, and then underwent the following behavior, biomarker, and MRI examinations.

### Behavior assessments

The behavior assessments conducted in this study could be classified into four domains, including cognitive, mental, physical, and fatigue.

#### Cognitive assessments

The cognitive assessments included two structured cognitive tests for global cognition, the MoCA [51, 52] and the cognitive subscale of the Alzheimer's Disease Assessment Scale (ADAS-cog) [53–55] in traditional Chinese. Additional tests for testing specific cognitive domains were the Digit Symbol Substitution Test (DSST), the Digit Span Test (DST), the Category Fluency (CF), the Letter-Number Sequencing (LNS), and the Facial Memory Test (FMT) from the Wechsler Adult Intelligence Scale-III [56].

#### Mental assessments

The MINI was used to exclude participants with psychiatric disorders [57]. We also used the Hospital Anxiety and Depression Scale (HADS) to evaluate the degree of anxiety (HADS-A) and depression (HADS-D) [58], as well as Hamilton Anxiety Rating Scale (Ham-A) and Hamilton Depression Rating Scale (Ham-D) [59, 60].

#### Physical activity evaluation

Quantification of daily physical activity utilized the International Physical Activity Questionnaire Short Form (IPAQ-SF), which asked the participants to report their physical activity by four generic items and transformed the degree and duration of activities into the metabolic equivalent of task minutes per week (MET). The four high-to-low-activity items were vigorous-intensity physical activity, moderate-intensity activity, walking, and sitting. In addition, the daily physical activity was also stratified into high, moderate, and low categories [61].

#### Fatigability assessment

The University of Pittsburgh developed the Pittsburgh Fatigability Scale (PFS) in 2015 to identify older adults at risk of mobility decline. The PFS measured mental and physical fatigability using a 10-item performance-based questionnaire on social, sedentary, lifestyle or light-intensity, and moderate to high-intensity activity-related fatigue [62]. We used the traditional Chinese version of PFS, which had been validated in Taiwan [63].

### Plasma biomarkers: the blood neurodegenerative proteins

We extracted plasm samples from peripheral venous blood in the ethylenediaminetetraacetic acid (EDTA) coated vacuumed blood tube and stored them at −80 ℃ until the immunomagnetic reduction (IMR) tests. IMR utilizes magnetic susceptibility changes upon antigen-antibody conjugation on magnetic nanoparticles. The novel technique uses a superconducting quantum interference device (SQUID) to detect the reduction of oscillation when the target protein is bound to the magnetic nanoparticle. IMR is capable of detecting trace concentrations of the target proteins in blood samples at a picogram level [64, 65]. At IMR assays, the plasma samples were mixed with IMR reagents for detecting Aβ42, Aβ40, Tau, p-Tau181, NfL, and GFAP separately (MagQu, Taiwan). The plasma concentrations for the above proteins were read by an IMR analyzer XacPro-S (MagQu, Taiwan) and duplicated for averaged results [66, 67].

### MRI acquisition

MRI data were obtained with a Siemens MAGNETOM Skyra 3 T MRI and 20-channel phased-array head/neck coil at Keelung Chang Gung Memorial Hospital in Taiwan. High-resolution anatomical images were acquired using MPRAGE T1-weighted imaging sequence in sagittal planes (TR/TE/TI = 2200/2.45/900 ms, flip angle = 8, acquisition matrix = 256 × 256, slice thickness = 1 mm, in-plane resolution = 1 mm^2^). Multi-shell dMRI data were acquired using the multi-band accelerated echo-planar imaging sequence (2.4-mm isotropic voxel, TR/TE = 8500/99 ms, multi-band acceleration factor = 4, phase encoding in the posterior-anterior direction, number of diffusion gradient directions = 30/64 at b = 1500/3000 s/mm^2^, respectively, each accompanied by one b = 0 image). An additional one b = 0 image volume was acquired with inverse phase encoding direction, specifically in the anterior-posterior direction, to correct image distortion.

### MRI data preprocessing

The dMRI data preprocessing steps included denoising [68], Gibbs ring removal [69], and correction for image distortion, inter-volume and slice-to-volume movement [70–72], and bias field [73]. Preprocessed dMRI data were up-sampled to 1.25 mm [74]. Finally, quality assessments were performed to exclude those with artifacts, excessive signal loss, or motion during the scan using FSL's tool for quality control [75]. All preprocessing steps for dMRI data were conducted using MRtrix3 [76], except for slice-to-volume motion correction, which was performed with FSL [77], and bias field correction, which was conducted using ANTs.

### FBA metrics and statistics

We followed the recommended FBA processing steps and parameters of MRtrix3, using multi-shell multi-tissue constrained spherical deconvolution to compute white matter FODs and tissue compartments of gray matter and cerebrospinal fluid [78]. Compartmental inhomogeneities were corrected via multi-tissue intensity normalization [79]. A study-specific FOD template was constructed from all the participants of the NC and SCD groups using the FOD-guided registration [80]. The FOD segmentation was then performed to produce template fixels, where each participant's fixel metrics were mapped onto. For each participant, we obtained the fixel-wise fiber density (FD), fiber-bundle cross-section (FC), and combined FD and FC (FDC). FD is proportional to the intra-axonal volume of specific white matter fiber bundles presenting within a voxel; FC reflects the macroscopic volumetric change of a local fiber bundle in the transverse plane relative to the FOD template; FDC, which estimates overall connectivity by computing the product of microscopic density and macroscopic cross-sectional area of a fiber bundle [30].

The whole-brain fixel-wise statistical analysis of these metrics was performed using a general linear model (GLM). This was achieved by generating a whole-brain tractogram on the FOD template, post-processing with spherical-deconvolution-based filtering of tractograms [81], and then computing fixel-to-fixel connectivity for the connectivity-based fixel enhancement (CFE) [82]. First, we performed categorical analysis to examine the differences in FBA metrics between the SCD and NC groups (Fig. 1B). Next, we conducted a dimensional analysis for SCD and NC two groups to investigate both the brain-biomarker and brain-behavior correlations (Fig. 1C). To investigate which fixel (dependent variable) in the brain could be predicted by each independent variable, mass-univariate GLMs were constructed separately for the whole sample.

For each GLM model, the nuisance covariates included participants’ age, gender, and educational years; intracranial volume was controlled additionally whenever applied to FC and FDC. All the variables were centered and normalized into a range from 0 to 1. The residuals were calculated as the difference between the observed values and the values predicted by the model, adjusted for the link function used in the GLM. For intergroup comparisons, nonparametric testing with 5,000 permutations was used for family-wise error (FWE) correction across multiple hypotheses to each fixel. The corrected FWE *p*-value (hereinafter *pFWE*) < 0.05 was considered statistically significant. In addition, considering the size of independent variables in the dimensional analysis, a more rigorous control for *pFWE* < 0.05 was performed alongside 10,000 permutations, hereinafter denoted as strong *pFWE* [83, 84].

## Results

### Participants demographics

As shown in Table 1, the final enrollment included 33 participants in the SCD group and 46 in the NC group. Both groups were comparable in age (68.06 ± 6.07 vs. 67.37 ± 3.28 years old,* p* = 0.555), sex (45.5 vs. 54.3%, *p* = 0.436), and education level (10.42 ± 4.76 vs. 9.96 ± 4.06 years, *p* = 0.640). There were no between-group differences in plasma Aβ42, Aβ40, Aβ42/Aβ40 ratio, total tau, NfL, and GFAP, only that plasma p-Tau181 was lower in SCD than NC group (3.76 ± 0.55 vs. 4.11 ± 0.45 pg/ml, *p* = 0.019). Compared to NC, the results of cognitive assessments showed that SCD had higher subjective cognitive concerns due to a higher AD8 score (3.85 ± 1.82 vs. 0.22 ± 0.42, *p* < 0.001) as well as inferior cognitive performance, as revealed by a higher ADAS-cog (7.48 ± 3.95 vs. 5.05 ± 3.12, *p* = 0.004) and a lower category fluency of color (11.76 ± 4.02 vs. 14.30 ± 4.27, *p* = 0.009) and FMT (34.39 ± 4.25 vs. 36.61 ± 5.14, *p* = 0.046). In addition, participants with SCD had a higher anxiety tendency with a higher HADS-A score (5.09 ± 3.96 vs. 2.93 ± 2.64, *p* = 0.005) (See Table 1 for the complete data).

**Table 1:** Demographic data, behavior indices, and blood-based biomarkers in SCD and NC

	SCD (N = 33)	NC (N = 46)	*p*
**A. Demographics**			
Age	68.06 ± 6.07	67.37 ± 3.28	0.555
Sex (female)	15 (45.5%)	25 (54.3%)	0.436^a^
Education	10.42 ± 4.76	9.96 ± 4.06	0.640
Married	40 (87.0%)	28 (84.8%)	0.790^a^
**B. Plasma biomarker**^†^			
Aβ42 (pg/ml)	16.29 ± 0.56	16.53 ± 0.42	0.093
Aβ40 (pg/ml)	53.42 ± 5.60	52.58 ± 7.55	0.665
Tau (pg/ml)	21.71 ± 2.93	23.19 ± 2.62	0.072
p-Tau181 (pg/ml)	3.76 ± 0.55	4.11 ± 0.45	0.019^*^
NfL (pg/ml)	9.70 ± 2.90	9.16 ± 2.76	0.514
GFAP (pg/ml)	17.42 ± 5.99	19.09 ± 6.90	0.376
Aβ42/ Aβ40	0.308 ± 0.035	0.321 ± 0.052	0.319
Tau/ Aβ42	1.334 ± 0.179	1.403 ± 0.155	0.161
p-Tau181/ Aβ42	0.231 ± 0.035	0.249 ± 0.028	0.057
**C. Cognitive assessment**			
AD8	3.85 ± 1.82	0.22 ± 0.42	< 0.001^*^
MoCA	24.91 ± 3.40	26.07 ± 3.45	0.144
ADAS-cog	7.48 ± 3.95	5.05 ± 3.12	0.004^*^
DSST	50.61 ± 19.88	57.65 ± 17.14	0.096
DST-f	11.73 ± 2.71	12.09 ± 2.29	0.525
DST-b	7.21 ± 3.14	6.87 ± 2.75	0.609
LNS	8.64 ± 2.91	9.41 ± 2.48	0.206
CF-animal	17.82 ± 4.43	19.78 ± 5.35	0.088
CF-fruit	12.58 ± 3.01	14.02 ± 3.44	0.056
CF-color	11.76 ± 4.02	14.30 ± 4.27	0.009^*^
CF-city	21.79 ± 5.77	20.89 ± 6.33	0.521
FMT	34.39 ± 4.25	36.61 ± 5.14	0.046^*^
**D. Physical activity**			
IPAQ-SF MET (min/week)	4668 ± 2581	5279 ± 3679	0.415
IPAQ-SF category (high,median,low)	25,6,2 (75.8,18.2,6.1%)	33,9,4 (71.1,19.6,8.7%)	0.888^a^
**E. Mental assessment**			
HADS-A	5.09 ± 3.96	2.93 ± 2.64	0.005^*^
HADS-D	4.88 ± 3.82	3.41 ± 3.40	0.077
Ham-A	3.58 ± 2.76	3.18 ± 3.48	0.589
Ham-D	3.18 ± 2.97	2.13 ± 2.50	0.095

F. Fatigue	SCD (N = 33)	NC (N = 46)	*p*	SCD (N = 33)	NC (N = 46)	*p*
Pittsburgh Fatigability Scale (PFS)	Physical			Mental		
PFS total score	18.70 ± 12.20	18.91 ± 10.31	0.993	15.76 ± 12.16	14.78 ± 10.42	0.703
PFS subitem						
A. Leisurely walk for 30 min	0.82 ± 1.24	0.69 ± 1.18	0.641	0.52 ± 1.00	0.42 ± 0.94	0.676
B. Brisk or fast walk for 1 h	2.06 ± 1.69	1.98 ± 1.63	0.828	1.48 ± 1.62	1.04 ± 1.40	0.203
C. Light household activity for 1 h	1.15 ± 1.44	1.24 ± 1.33	0.770	1.06 ± 1.52	1.02 ± 1.22	0.902
D. Heavy gardening or outdoor work for 1 h	2.48 ± 1.77	2.80 ± 1.63	0.419	2.15 ± 1.86	2.00 ± 1.80	0.718
E. Watching TV for 2 h	0.85 ± 1.20	1.11 ± 1.21	0.345	0.91 ± 1.23	0.84 ± 1.13	0.811
F. Sitting quietly for 1 h	0.79 ± 1.17	0.73 ± 1.20	0.841	0.79 ± 1.17	0.76 ± 1.23	0.905
G. Moderate-to-high-intensity strength training for 30 min	3.33 ± 1.78	3.31 ± 1.55	0.953	2.70 ± 1.85	2.69 ± 1.87	0.985
H. Participating in a social activity for 1 h	1.00 ± 1.30	0.69 ± 0.97	0.230	1.03 ± 1.38	0.76 ± 1.03	0.316
I. Hosting a social event for 1 h	1.58 ± 1.70	1.44 ± 1.53	0.722	1.52 ± 1.70	1.60 ± 1.70	0.828
J. High-intensity activity for 30 min	2.27 ± 1.76	2.36 ± 1.57	0.827	1.58 ± 1.62	1.69 ± 1.59	0.759

^a^A chi-square test for categorical variables; other variables were examined with independent t-test

^†^A total of 23 SCD and 25 NC participants had complete plasma biomarker tests

^*^A *p* < 0.05 for statistical significance

### Categorical comparisons

**Fig. 2:**
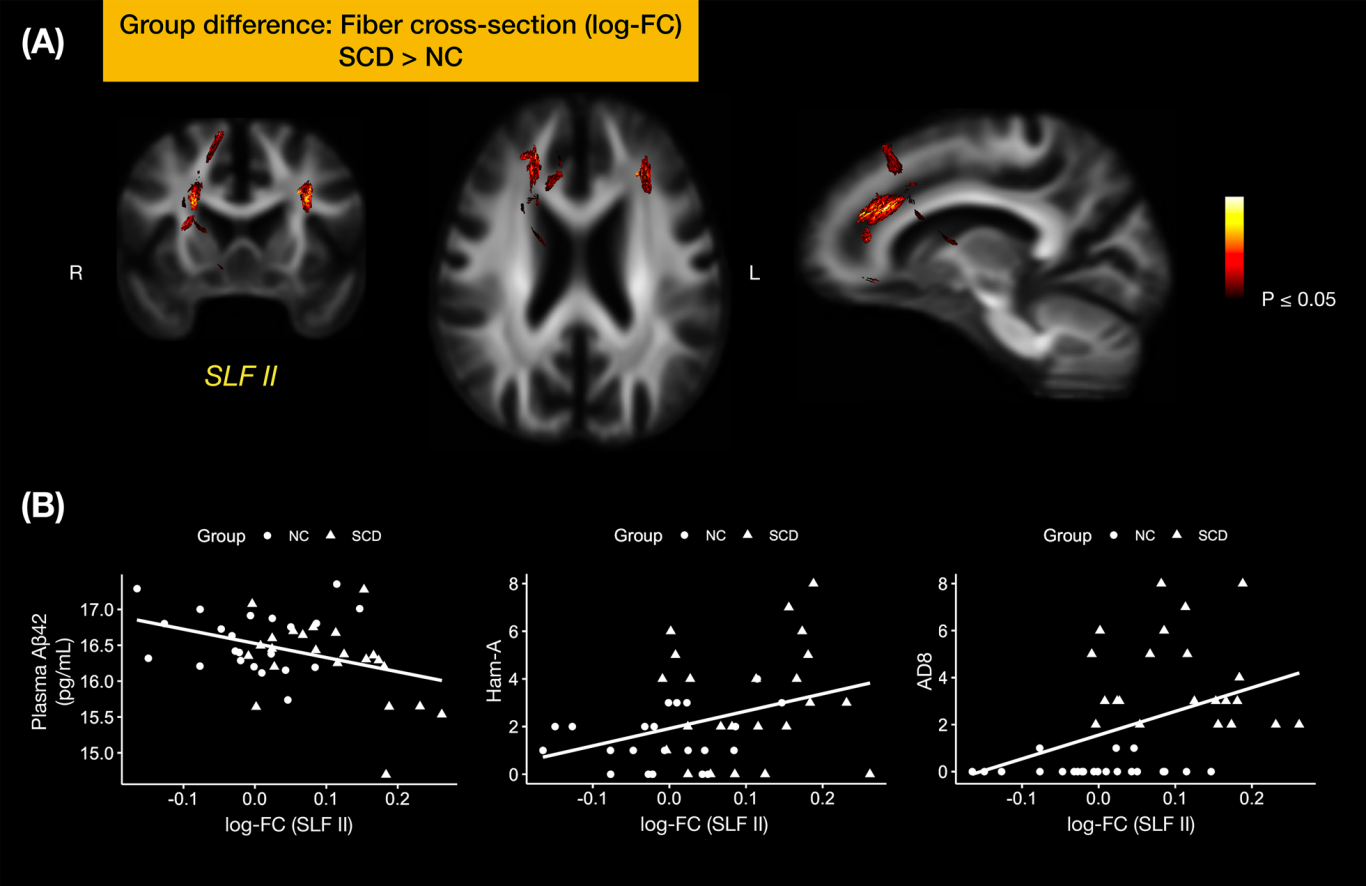
Fiber-bundle cross-section comparison of NC and SCD. **A:** For fixels that reached statistical significance (pFWE < 0.05), we mapped the fixel-wise pFWE onto the associated streamlines, showing white matter tract segments where SCD had significantly greater log-FC than NC (pFWE < 0.05). **B** The log-FC of these white matter regions (i.e. fixels) correlated with behavior metrics and blood biomarkers, including blood Aβ42 (left), the anxiety degree by Ham-A score (middle) and the degree of subjective cognitive complaints by AD8 score (right)

Compared to the NC, the SCD group had a significantly larger FC at bilateral frontal SLFII (*pFWE* < 0.05) (Fig. 2A). There were no statistically significant differences in FD and FDC metrics between the SCD and NC groups.

### Dimensional relationships between brain, plasma-biomarker, and behavior

**Fig. 3:**
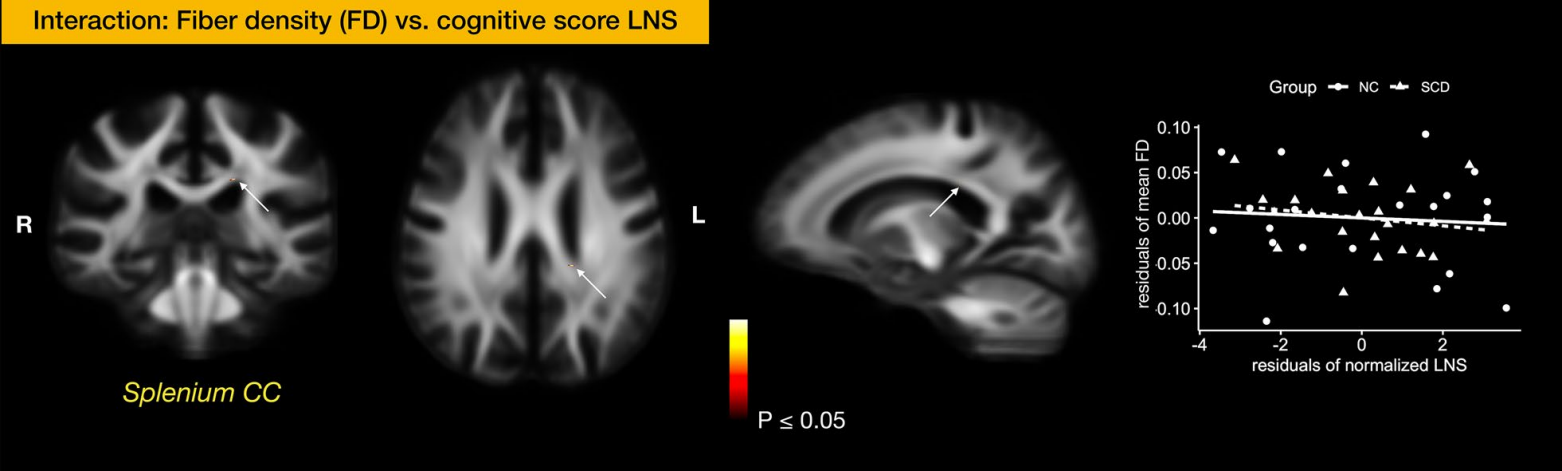
Interaction of SCD between the cognitive score and FBA metrics. SCD grouping interacted with the score of Letter-Number Sequencing and the FD at the splenium of corpus callosum. The interaction effect was significant at a* p* < 0.05 under strong permutation for family-wise error (FWE) correction across multiple hypotheses

**Fig. 4:**
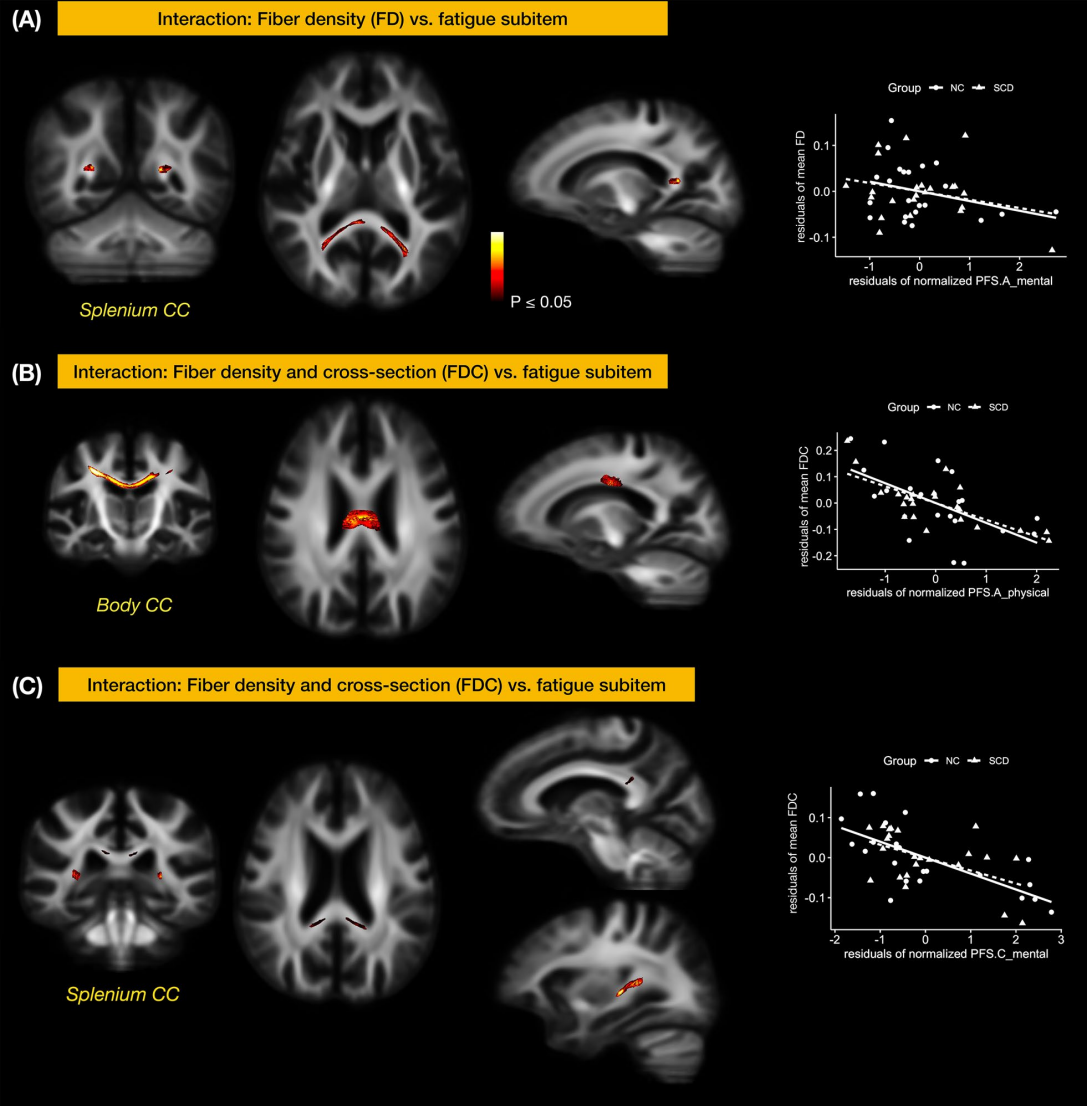
Interaction of SCD between the Pittsburg Fatigue Scale subitems and FBA metrics. Neither the correlations of the total physical fatigability and FBA metrics nor the correlations of total mental fatigability and FBA metrics differed between SCD and NC. However, SCD grouping interacted between several fatigability subitems and FBA metrics in different regions. **A:** SCD grouping interacted between a fatigability subitem and the FD of the splenium of corpus callosum; the fatigability subitem was the mental fatigability for a leisurely walk for 30 min (PFS.A_mental). **B:** SCD grouping also interacted between the FDC of the body of corpus callosum and the physical fatigability for a leisurely walk for 30 min (PFS.A_ physical). **C:** Besides, SCD and NC groups also showed different correlations for the mental fatigability for doing light household activities for 1 h (PFS.C_mental) and the FDC metrics of fiber tracts of the splenium of corpus callosum. The interaction was significant at a *p* < 0.05 for a strong family-wise error (FWE) control

- Whole-brain FBA metrics versus plasma biomarkers* − *Under strong FWE correction, there was no significant interaction of SCD grouping in correlations of FBA metrics and plasma biomarkers.
- Whole-brain FBA metrics versus behavior measures *− *In the cognitive domain, we found the interaction effects between LNS and FD at the splenium of corpus callosum (strong *pFWE* < 0.05, 7 fixels) between SCD and NC (Fig. 3). In the fatigue domain, there were significant interaction effects at several brain regions either with the FD or FDC, even though the total scores for mental and physical fatigability were not statistically different between the SCD and NC groups. The interaction between the mental fatigability for a leisurely walk for 30 min (PFS.A_mental) and FD at the splenium of corpus callosum (strong *pFWE* < 0.05, 128 fixel) was found (Fig. 4A). The FDC at the body of corpus callosum (strong *pFWE* < 0.05, 978 fixels) was negatively correlated with the physical fatigability for a leisurely walk for 30 min (PFS.A_physical) (Fig. 4B). Splenium of corpus callosum from FDC metrics showed a negative correlation with the mental fatigability for doing light household activities for 1 h (PFS.C_mental) (strong *pFWE* < 0.05, 183 fixels) (Fig. 4C). In the mental and physical domains, there were interaction effects with FBA metrics, but none of them passed the strong FWE correction.
- Fixel-wise FC at SLFII versus plasma biomarkers/behavior data* − *From the whole-brain dimensional analysis (i.e. a) and b) above), we did not identify any associations of SLFII and biomarkers/behavior data. To this end, we further extracted the mean log-FC values from SLFII, and found that the mean log-FC of SLFII correlated negatively with plasma Aβ42 level (*r* = −0.38, *p* = 0.01) and positively with AD8 (*r* = 0.42, *p* = 0.004) and Ham-A (*r* = 0.35, *p* = 0.019) (Fig. 2B).

## Discussion

Using multi-shell dMRI data acquisition and FBA, we found SCD-associated structural changes in white matter. Relative to NC, adults with SCD showed increased macroscopic fiber-bundle cross-section at bilateral frontal SLFII. Also, cognition and anxiety symptoms and plasma Aβ42 level were correlated with SLFII, suggesting that macrostructural alterations in this frontal region may be crucial to the development of preclinical dementia. In addition to the altered SLFII, the white matter structure at the splenium of the corpus callosum and right thalamus was found to dimensionally link with plasma NfL. In search of the impacts of SCD on FBA-multidomain correlations, the microstructure of corpus callosum appeared stronger associations with the LNS cognitive score and weaker associations with fatigue to leisure activities in SCD than NC. These findings suggest that SCD is associated with the altered associations between behavior and callosal white matter fibers.

### SLFII: implications for SCD

Originated from the angular gyrus and inferior parietal lobule (IPL), SLFII projects anteriorly into the ipsilateral superior frontal gyrus and middle frontal gyrus [85, 86]. In both hemispheres, SLFII is involved in motor control and working memory. However, functional lateralization of SLFII is commonly known, where the right SLFII is responsible for maintaining visuospatial function, and the left SLFII is responsible for tool use and language function [87, 88]. Consistent with previous studies using DTI [15, 17–19], we found SCD-associated alterations in the SLFII tracts, which might be relevant to motor planning, visuospatial attention, working memory, or language processing impairments at a crucial stage to initiate the dementia continuum. We also found that the region showing such intergroup differences linked dimensionally with the LNS cognitive test score, which is an indicator of executive function involving working memory as well as attention and visuospatial coordination [89]. This brain-cognition correlation echoes the importance of SLFII-related executive function in preclinical dementia [88].

**Fig. 5:**
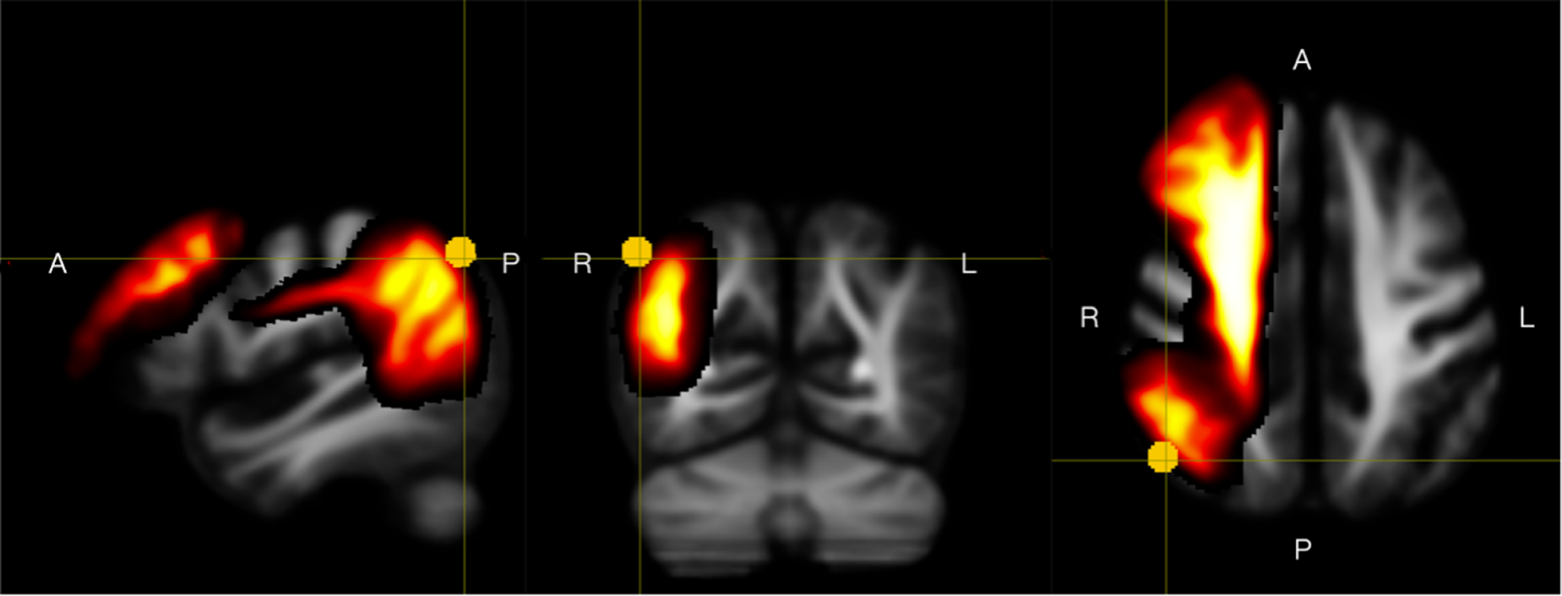
Anatomical overlap of right IPL and SLFII. Comparing the regions of interest (ROIs) with local dynamic connectivity di﻿fference [55] and the fixels with FBA metrics of fiber cross-section difference between SCD and NC, the right inferior parietal lobe ROI (yellow dot) [91] is anatomically co-localized with the SLFII fiber bundles (red-gradient area) [90]. The methods of generating this figure are available in Supplementary 1

In relation to our previous results from functional MRI, we also found that the spatial distribution of SLFII (as provided by a white matter atlas [90, 91]) overlapped with the area with functional connectivity differences within the same population (Fig. 5), suggesting a functional–anatomical coupling. Our previous study revealed significant decreases in local dynamic functional connectivity at the right IPL in SCD compared to normal controls [55]. Because the IPL is at the origin of SLFII, the functional connectivity decreased at IPL in SCD might be linked with the macrostructural changes at SLFII. However, even though these two regions were anatomically overlapped, our further analysis did not show a significant correlation between the mean dynamic amplitude of low-frequency fluctuation (mdALFF) value at right IPL and the log-FC at bilateral SLFII. In a previous study, although white matter alterations at SLF were shown to positively correlate with the functional connectivity of left retrosplenial cortex and left dorsomedial prefrontal cortex [19], the results could potentially be compromised by the use of tensor-based metrics. Therefore, future works are required to clarify functional-structural relationships for the early cognitive changes in the preclinical dementia SCD stage.

### Dystrophy/swelling of SLFII in SCD

Our study revealed that the bilateral frontal SLFII have higher fiber-bundle cross-sections in SCD compared to NC, rather than reductions as observed commonly in neurodegenerative disorders with FBA (e.g. [35]). We infer that the enlargement of SLFII's cross-section could potentially be a feature in the SCD population for the reasons below.

Within SCD, our data also demonstrated that the fiber cross-section enlargement at SLFII correlated dimensionally with a lower plasma Aβ42. This finding was consistent with amyloid-mediated axonopathy, in which amyloid plaque can initiate axonal dystrophy [92]. According to long-term in vivo investigations of transgenic mice, about a quarter of the neurites surrounding extracellular Aβ deposits exhibited early swelling and tortuous neuronal processes; such dystrophic changes coincided with synaptic alteration, followed by neurodegeneration [92]. The postmortem human pathohistological study also confirmed early axonal dystrophy and its association with amyloid pathology in early AD patients [36]. Additionally, the frontal lobe is also the area of initiation of amyloid plaque staging of AD [93, 94]. Therefore, the amyloid-axonopathy correlation in SCD could be the initial changes in the AD continuum.

At the ultrastructure of the Aβ-provoked axon swelling sites, there were degenerative components like abnormal micro-tubule-associated proteins, molecular motor proteins, organelles, vesicles, and mitochondria [37]. As a common hypothesis of amyloid-mediated axonopathy, axonal transport dysfunction could be multifactorial [37], involving amyloid-related axonal motor protein kinesin dysfunction, phosphorylated tau-induced microtubule destabilization and adaptor protein dysfunction, and mitochondrial dysfunction [95, 96]. Using the AD mouse model, the synergic impact of amyloid and tau on axonopathy was further confirmed in the AD mouse model by reducing tau levels to alleviate Aβ-induced axonal transport dysfunction [97]. However, while the underlying mechanisms relevant to Aβ-induced axonopathy were demonstrated in animal models, further studies are required to dissertate our findings of Aβ-associated axonal swelling in SCD in human brains.

### SCD-related SLF changes in the AD continuum

The aforementioned dimensional relationship between SLFII's enlargement in fiber-bundle cross-section and decrease in plasma Aβ42 might reflect axonal swelling and dysfunction in axonal transport, both of which could begin in the preclinical stages of the AD continuum. AD is associated with the buildup of Aβ plaques and tau protein tangles in the brain [98, 99], leading to disruptions in signal communication between different brain areas [100, 101]. A recent PET/dMRI study that included the full spectrum of biomarker-confirmed AD patients showed the presence of amyloid deposition, regardless of tau deposition, was linked to a decrease in both fiber density and cross-section metrics of FBA [34]. These findings support the presence of amyloid-focused damage to white matter fibers in the progression of AD. On the other hand, Mito and colleagues discovered a reduction in white matter specific to certain fibers in AD patients using FBA, as compared to patients with MCI and healthy controls [35]. They found that long-association white matter pathways such as SLF and cingulum were dominated by decreases in macrostructural fiber-bundle cross-section, whereas commissural and short-association fibers were mainly linked with reductions in microstructural fiber density. Interestingly, the bilateral SLF showed decreases specifically in fiber cross-section, suggesting white matter atrophy of these fiber tracts in AD patients. In the current study, we found white matter dystrophy at bilateral SLFII (as indicated by an increased log-FC metric) in adults with SCD. The changes in FC reflect macroscopic morphological changes orthogonal to bundle orientation [84]. We infer that such macrostructural dystrophy of white matter fibers might indicate an early stage of the full neurodegenerative processes − a progression of axonopathy from early swelling to late atrophy, as evidenced by the amyloid deposition-induced serial axonal dystrophy and loss observed in transgenic AD mouse models over time [92]. However, it may not be straightforward to validate the effects of axonal fiber swelling on the FC metric since the FC metric of FBA is computed based on the warp field obtained from the FOD-based registration. Biophysical modeling and simulation may be required for such an investigation, which is nevertheless beyond the scope of the current study.

### Splenium of corpus callosum: implications for SCD

The splenium of the corpus callosum is comprised of compact fiber bundles interconnecting bilateral temporal-occipital regions for visuospatial, language, and behavior coordination [102]. Injuries to this region can cause disturbances of consciousness, hallucinations, psychosis, and disconnection syndrome with apraxia, alien hand, alexia, and agraphia [103]. Previous studies showed SCD-related white matter structural changes at the splenium of the corpus callosum, including lower FA [16–18] and callosal atrophy [104]. The splenium section was also found to be crucial for an effective cognitive training response in SCD [105].

In this study, although there were no significant differences in FBA metrics between our SCD and NC groups at the corpus callosum, we found that the FD and FDC metrics at the splenium section were associated dimensionally with the behavior presentation of SCD. First, FD at the splenium showed stronger negative correlations with working memory (as indexed by the LNS test) in SCD than NC, suggesting that the subtle cognitive decline in SCD was associated with callosal white matter. Second, FDC at both the splenium and body showed weaker negative correlations with mental and physical fatigue in daily activities in SCD than NC, implying that SCD might be a general condition not bonded to callosal injury. Both findings indicate the important role of the corpus callosum in SCD-related multidomain changes.

Based on our study population, no significant associations were identified between FBA metrics and plasma biomarkers under the strong FWE correction. Nevertheless, it might be worth mentioning that the FD metric at the splenium section positively correlated with plasma NfL with the regular FWE control (Figure S1). This might suggest that the high-density callosal fibers at the splenium section were the major supply of plasma NfL, which is usually released into plasm upon axonal injuries. However, it remained homeostatic and did not step into the imbalanced axonal degeneration and cognitive decline in SCD.

### Limitations

First, the study did not recruit SCD participants from memory clinics, meaning that our SCD cohort might mainly consist of participants with early cognitive decline (i.e. early SCD), rather than those closer to MCI (i.e. late SCD) who may have more intense white matter changes. Hence, our results might not be generalizable due to the limited coverage of people with SCD. Also, the number of enrolled participants was limited. Increasing the sample size and range could increase the statistical power and generalizability of our findings. Second, our inferences in the Discussion above were drawn based on SCD considered an early indicator of AD; however, not all individuals with SCD will develop AD. Studies have shown that a significant proportion of individuals with SCD do not progress to MCI/AD [3, 106]. Some individuals with SCD may experience a cognitive decline due to other factors, such as vascular risk factors. Third, SCD is typically based on self-reported symptoms only, which may not accurately reflect the presence or severity of the underlying cognitive impairment. The use of diverse diagnostic criteria for SCD across studies can complicate the comparisons between studies, leading to challenges in drawing decisive conclusions. SCD itself may not be sufficient for predicting future AD conversion, and additional biomarkers and assessments are needed to improve diagnostic accuracy. Therefore, further FBA studies to include different stages of the dementia continuum are warranted to reveal sequential white matter structural changes. Finally, although our dMRI protocol opted for a maximum b-value of 3000 s/mm^2^ to suit a clinical MRI scanner with standard gradient strength, even higher b-values have been suggested as beneficial for FBA [107]. Additionally, given the fundamental requirement of using high b-values (≥ 3000 s/mm^2^) to represent intra-axonal volume for FD [108], further studies are warranted to assess the implications of incorporating low b-values (< 3000 s/mm^2^) into multi-shell multi-tissue constrained spherical deconvolution; such inclusion of low b-values in multi-tissue analyses might affect FD estimations [107].

## Conclusion

Axonal dystrophy in the brain is known to be one of the earliest morphological changes associated with cognitive decline. The present study showed that people with SCD had greater fiber-bundle cross-sections at bilateral frontal SLFII than NC, and such macrostructural enlargement of SLFII correlated with amyloid pathology, presentation of anxiety, and degree of subjective cognitive worries. There were also anatomical overlaps between SLFII and alterations in local functional connectivity at the IPL, suggesting joint structural-functional changes in SCD. In addition, white matter fiber density at the splenium of the corpus callosum was associated with cognitive performance as well as mental and physical fatigability of the SCD subjects. This demonstrated the importance of cross-hemisphere connections in early cognitive decline of dementia continuum. Future longitudinal follow-up studies will be needed to depict white matter degeneration trajectories, to answer whether bilateral SLF dystrophic swelling could turn into atrophy, and whether plasma Aβ42 and NfL could be promising biomarkers for axonal fiber degeneration and cognitive decline.

### Supplementary Information

Supplementary material 1.

Supplementary material 2: Figure S1. Correlations of biomarker and FBA metrics in all enrolled participants. Plasma NfL positively correlated with FD at the splenium of corpus callosum (*pFWE*<0.05, 12 fixel) and with log-FC at the right thalamus (*pFWE*<0.05, 6 fixel).

## Data Availability

The datasets generated and/or analyzed during the current study are available from the corresponding author upon reasonable request.
