# Supplementary material for: White matter alterations and their associations with biomarkers and behavior in subjective cognitive decline individuals: a fixel-based analysis
Source: Behav Brain Funct. 2024 May 22;20:12. doi: 10.1186/s12993-024-00238-x (PMC11110460; doi:10.1186/s12993-024-00238-x)
Supplement: Supplementary file 1 — Supplementary material 1. [file 12993_2024_238_MOESM1_ESM.pdf]

Supplementary 1: The methods of generating Figure 5.

a) For each participant, we conducted diffusion tensor analysis to generate a fractional anisotropy (FA) map, using only the "inner shell" dMRI data (30 directions at b=1500 s/mm^2).

b) Subsequently, the participants' FA maps were transformed to the group FOD template space using their respective transformation functions (both linear and non-linear), which were obtained during the generation of the population template.

c) A group FA template was then generated by averaging all participants' FA maps. It is worth noting that this FA template was aligned with the FOD template.

d) Following this, we used ANTs (command antsRegistration) to conduct registration between our group FA template and the FA template from the Human Connectome Project dataset (available in FSL, file name: FSL_HCP1065_FA_1mm.nii.gz). This step yielded transformation functions between our population template space and the standard MNI space.

e) Finally, the right inferior parietal lobe ROI (derived from our prior fMRI analysis on the same cohort) and the superior longitudinal fasciculus (SLFII; sourced from a white matter atlas [90]) were both mapped from the MNI space to our template space.
