# Supplementary material for: White matter alterations and their associations with biomarkers and behavior in subjective cognitive decline individuals: a fixel-based analysis
Source: Behav Brain Funct. 2024 May 22;20:12. doi: 10.1186/s12993-024-00238-x (PMC11110460; doi:10.1186/s12993-024-00238-x)

Figure S1: Correlations of biomarker and FBA metrics in all enrolled participants. Plasma NfL positively correlated with FD at the splenium of corpus callosum (*pFWE*<0.05, 12 fixel) and with log-FC at the right thalamus (*pFWE*<0.05, 6 fixel).

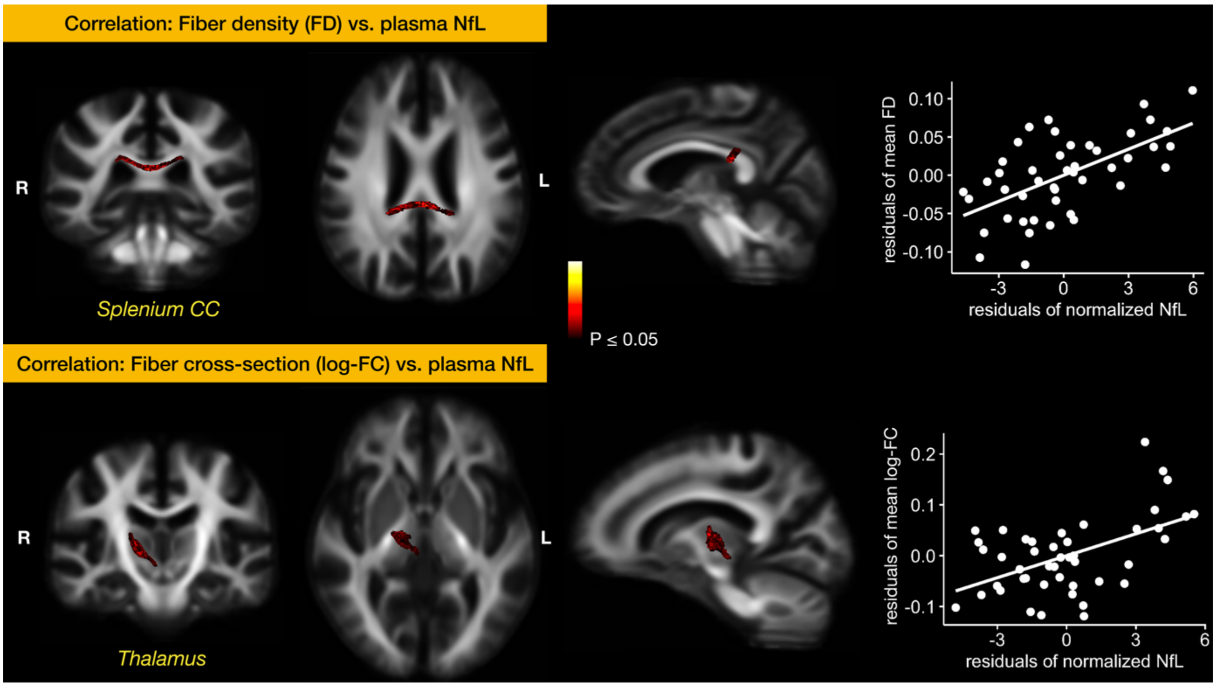

Supplement: Supplementary file 2 — Supplementary material 2: Figure S1. Correlations of biomarker and FBA metrics in all enrolled participants. Plasma NfL positively correlated with FD at the splenium of corpus callosum (pFWE<0.05, 12 fixel) and with log-FC at the right thalamus (pFWE<0.05, 6 fixel). [file 12993_2024_238_MOESM2_ESM.pdf]
